# Skeptical Arguments and Deep Disagreement

**DOI:** 10.1007/s10670-021-00433-6

**Published:** 2021-06-25

**Authors:** Guido Melchior

**Affiliations:** Department of Philosophy, University of Graz, Heinrichstrasse 26/5, 8010 Graz, Austria

## Abstract

This paper provides a reinterpretation of some of the most influential skeptical arguments, Agrippa’s trilemma, meta-regress arguments, and Cartesian external world skepticism. These skeptical arguments are reasonably regarded as unsound arguments about the extent of our knowledge. However, reinterpretations of these arguments tell us something significant about the preconditions and limits of persuasive argumentation. These results contribute to the ongoing debates about the nature and resolvability of deep disagreement. The variety of skeptical arguments shows that we must distinguish different types of deep disagreement. Moreover, the reinterpretation of skeptical arguments elucidates that deep disagreement cannot be resolved via argumentation.

## Skeptical Arguments

Typically, epistemologists identify two desiderata concerning skeptical arguments, explaining how the skeptical intuition arises and showing why the skeptical argument is unsound (or why it is sound). Most epistemologists do not regard skeptical arguments as convincing. In particular, they do not find regress arguments cogent, and consequently, they reject them as arguments about the limits of knowledge. In discussions of skeptical arguments, it is occasionally pointed out that we are not in a position to present a persuasive argument to the skeptic but that this does not imply that we cannot know.^1^ These authors mainly aim to provide a counterargument against the skeptic, i.e. they intend to show why the skeptical argument fails despite seeming so convincing. Approaches to skeptical problems usually stop at this point due to the strong focus on the concept of knowledge in epistemology. Even if the skeptical conclusion can be reasonably rejected, however, skeptical arguments can still provide insights about persuasive argumentation. The core idea of this paper is that the problems and limitations of reasoning stressed by these arguments do not affect our capacities to know but our capacities to persuasively argue. Properly understood, these arguments do not teach a lesson concerning the limits of knowledge but concerning the limits of persuasive argumentation. Hence, they are unsound as skeptical arguments about the extent of knowledge, but, properly reinterpreted, they are sound arguments about the limits of persuasive argumentation.

Argumentation might seem a rather neglected phenomenon in contemporary epistemology, despite the raising popularity of social epistemology, but this impression is not entirely correct, given the substantial recent literature on argumentation in epistemology.^2^ Moreover, argumentation theory as a stand-alone discipline reflects on the epistemic aspects of argumentation.^3^ Argumentation theorists also stress the importance of common ground for persuasive argumentation.^4^ However, there is no thorough examination on the market in the field of argumentation theory that classifies the preconditions of persuasive argumentation and the limits of establishing common ground via argumentation along the lines of skeptical arguments, as I will argue for in this paper. Furthermore, many of the views presented here have been informally introduced in the philosophical area of epistemology in discussions of skeptical problems. Hence, there is notable preliminary work from various angles concerning the central views defended here, but it lacks a systematization and a thorough application to the problem of deep disagreement, which is provided in this paper.

In this paper, I will investigate two argumentative phenomena—argumentation with a skeptic (instead of argumentation for or against skepticism) and deep disagreement. I will focus on the following three skeptical arguments:

1. Agrippa’s trilemma
2. A meta-regress argument
3. The argument for external world skepticism, including the claim that Moorean reasoning is epistemically defective because it cannot offer a persuasive argument to the skeptic.

This list of skeptical arguments is, of course, not exhaustive. For example, there are other explanations on the market of why Moorean reasoning is epistemically defective.^5^ Hence, I do not claim that any skeptical argument can be reinterpreted as an argument about the limits of persuasive argumentation, however a substantial subclass can be. Arguments (A1)–(A3) rely on the implicit intuition that we cannot have knowledge because the possibilities of reasoning are limited. (A1) is based on the idea that we cannot perform infinite reasoning, and that neither circular reasoning nor stopping a reasoning process without providing further reasons can yield knowledge; (A2) on the idea that we cannot acquire knowledge because we cannot perform an infinite meta-regress of reasoning; (A3) on the claim that we cannot know via Moorean reasoning because it cannot convince a skeptic.

Before proceeding, let me pause for a moment to say a few words about the structure of the skeptical arguments that will be analyzed. (A1) and (A2) are regress arguments, i.e. they rely on a regress clause which implies that one has to undergo infinitely many steps for achieving justification and/or knowledge, a requirement that cannot be fulfilled. (A1) is based on the idea that justification or knowledge of *q* requires justification via another proposition *p* which itself has to be justified and so on. (A2), in comparison, relies on the assumption that in order to know (or be justified to believe) that *q* on the basis of *p*, S needs to know (or be justified to believe) that *p* properly supports *q*. Note that there are not only structural similarities between (A1) and (A2). Carroll (1895) presents in his famous essay ‘What the tortoise said to Achilles’ a version of the regress argument which can be regarded as hybrid of (A1) and (A2). He suggests that in order to demonstrate that *q* via inference from *p*, you have to add as premise that if *p* is true, then *q* is true, and in order to demonstrate that *q* on the basis of this set of premises you have to add as premise that if these premises are true, then *q* is true and so on. Thus, in order to demonstrate that *q*, you have to prove infinitely many premises, which is impossible.^6^ In Carroll’s argument, the premises that have to be added are about the relation between premises and conclusion and, therefore, have the same content as meta-arguments in (A2), but they are incorporated as premises of the argument for *q*, and consequently are not literally meta-arguments. This example shows that (A2) can be transformed into an argument that is at least close to (A1). However, I do not see how (A1) could be formulated as a version of (A2). Furthermore, I think there is an intuitive difference between requiring an argument for every premise used and requiring an argument for the adequacy of every argument presented. People often require further information about the premises of an argument, but asking for a meta-argument seems intuitively a different issue.^7^

Moreover, argument (A3) concerning Moorean reasoning and bootstrapping can also be interpreted as a regress problem. If S cannot come to know that a source is reliable via information delivered from the source itself, then S needs a second source for determining the reliability of the source and so on. Hence, an infinite regress threatens. However, this interpretation of (A3) ignores that there remains a puzzle about why bootstrapping and Moorean reasoning are or seem implausible, given that one rejects this kind of regress. Hence, all three arguments (A1)–(A3) are presumably interpretable as regress-arguments. Nevertheless, there are notable differences between these arguments, which are also crucial for revealing different types of deep disagreement. For these reasons, I will stick hereinafter to the useful distinction between (A1), (A2) and (A3).^8^

## Deep Disagreement

Let me start with some remarks about disagreement in general. Two parties disagree about a target proposition if they have different doxastic attitudes towards this proposition, i.e. if A believes that *p* and B rejects that *p* or suspends judgment about *p*.^9^ Disagreement is resolved when the two parties who initially disagree about *p* end up having the same doxastic attitude. One intuitive way of resolving disagreement is via persuasion. Hereinafter, I understand *persuasion* in the following sense:

**Definition: Persuasion**

A persuades B that *p* via action *c* iff A performs *c* and B believes that *p* because of *c*.^10^

Persuasion is an instance of resolving disagreement given that one party who believes that *p* makes another party also believe that *p*. This is the instance of resolving disagreement on which I will focus in this paper. Moreover, disagreement can be resolved via various methods, e.g. via demonstration. One paradigmatic way of resolving disagreement, which is subject of this paper, is via argumentation.

Let me now come to *deep* disagreement. In his seminal paper, Fogelin (1985) introduced the notion of deep disagreement to describe a phenomenon that has recently come back into the focus of social epistemology. Deep disagreement is intuitively introduced rather than precisely defined. Intuitively, it occurs if two parties fail to reach agreement for some fundamental and systematic reasons and not due to some obvious epistemic vices such as stubbornness. In this paper, I will adopt this intuitive understanding of deep disagreement. According to this understanding, not all irresolvable disagreements are deep. Fogelin (1985, p. 8)^11^ notes: “I can argue myself blue in the face trying to convince you of something without succeeding. The explanation might be that one of us is dense or pig-headed.” These are instances of irresolvable disagreement that are not deep in any interesting sense. As for deep disagreement, we can identify two core questions:

- Q1: What is deep disagreement?
- Q2: Is deep disagreement resolvable or not?

Different theories are defended concerning Q1. Fogelin (1985) opts for a Wittgensteinian take when he claims that deep disagreement “proceeds from a clash in underlying principles” and that disagreement is deep insofar as it concerns “a whole system of mutually supporting propositions (and paradigms, models, styles of acting and thinking) that constitute […] a form of life.”^12^ Aikin (2021a) provides an explanation of deep disagreement that is based on regress problems and the problem of the criterion. Lynch (2010, 2016) presents a theory of deep disagreement that is based on fundamental epistemic principles and which is connected to the bootstrapping problem as discussed here.^13^

There are also different takes on Q2. We can distinguish pessimistic views, which argue that deep disagreement cannot be rationally resolved, from optimistic views, which claim that there is a rational solution. Fogelin (1985) is a pessimist when he argues that “deep disagreements cannot be resolved through the use of argument, for they undercut the conditions essential to arguing.” Lynch (2010, p. 273) is also pessimistic when he metaphorically suggests: “Where there is deep epistemic disagreement over some fundamental principle, the disagreement has hit bedrock, the spade has turned.” However, there are also optimistic views concerning resolvability of deep disagreement. Feldman (2005) argues in response to Fogelin that deep disagreement is rationally resolved when both parties end up suspending judgment concerning the propositions about which they initially disagreed.^14^

This paper uses skeptical arguments to clarify both questions concerning deep disagreement. First, it clarifies what deep disagreement is by providing a taxonomy of various versions of deep disagreement. Second, it provides sound arguments for why these versions of deep disagreement are irresolvable, which are based on reinterpretations of skeptical arguments.

## Agrippa’s Trilemma

The first skeptical argument, discussed here, is Agrippa’s trilemma.^15^ As a skeptical argument for the claim that we cannot have any knowledge, it can be spelled out as follows:

**Agrippa’s trilemma**

1. There are exactly three potential alternatives of justification for believing that *p*:
  (i) foundationalist justification
  (ii) circular justification
  (iii) infinite justification
2. None of these three alternatives can yield justification for believing that *p*.
3. There is no knowledge without justification.
4. Therefore, we do not have any knowledge.

There is wide agreement in contemporary epistemology that the skeptical argument based on Agrippa’s trilemma is unsound since (2) is false because either (i), (ii), or (iii) can yield justification. Foundationalism accepts (i), coherentism (ii), and infinitism (iii) as a way of acquiring justification.^16^

Agrippa’s trilemma states that a subject is, under certain circumstances, forced to either reason ad infinitum, to stop at an arbitrary point, or to undergo circular reasoning. However, when would a subject face a trilemma of presenting new reasons ad infinitum, stopping at an arbitrary point, or reasoning circularly if not in cases of justification? The answer can be found in the context of argumentation, or so I will argue.^17^

Before making this point, let me provide some terminological remarks on argumentation and persuasion. I understand argumentation as an interaction between a speaker, to whom I refer as A, and a hearer, to whom I refer as B. One central purpose of argumentative interaction is to convince or persuade someone, i.e. to make someone believe a certain target proposition.^18^ Often, a speaker makes a hearer believe that *p* simply by uttering that *p*, without providing any further evidence for *p*.^19^ This is only possible if the hearer trusts the speaker in some sense. Accordingly, I define persuasion via trust as follows:

**Definition: Persuasion via Trust**

A persuades B via trust that *p* iff A utters that *p* and B believes that *p* because of A’s utterance that *p*.^20^

Persuasion via trust does not require that B reflectively believes that A is reliable or trustworthy concerning *p*. It can also happen rather automatically, for example, if a child automatically believes her parents without entertaining any thought about their reliability or trustworthiness. Hence, persuasion via trust often only involves the absence of a negative belief that the speaker is unreliable or lacks trustworthiness. For the purpose of this paper, I am particularly interested in persuasion via argumentation in the following sense:

**Definition: Persuasion via Argumentation**

A persuades B via argumentation that *p* iff A presents an argumentation for *p* and B believes that *p* because of this argumentation.^21^

Arguments consist of one or more premises and a conclusion.^22^ Belief formation is a psychological phenomenon, and hearers can believe the conclusion of an argument under various circumstances. Rationally, and presumably also usually, hearers believe the conclusion of an argument because of an argumentation only if they also believe the argument’s premises (or at least sufficiently many of them.) However, hearers occasionally violate these standards for rationality. For example, a hearer might fall prey to wishful thinking and, for this reason, believe the conclusion of the argument because of the argument although she does not believe its premises. However, we can hardly formulate any systematic account of persuasive argumentation if we take this kind of irrationality into account. Therefore, I am in this paper primarily interested in subjects for whom the following rationality principle PCB holds:

**PCB**

B believes that *c* because of A’s argumentation consisting of premises *p*_1_…*p*_n_ and conclusion *c*, only if B believes sufficiently many of premises *p*_1_…*p*_n_.

I will call hearers who follow PCB *unprejudiced*, since they do not believe the conclusion of an argument for merely psychological reasons and without believing the premises.^23^ Let us now consider conditions for persuasive argumentation. Given that B is unprejudiced, A will persuade B via argumentation only if B believes the premises of the argument presented.^24^ Thus, A has the following options to persuade B via argumentation: (1) B already believes sufficiently many of the presented premises prior to being confronted with them by A. (2) B trusts A concerning the premises, i.e. B believes the premises because of A’s utterance. (3) B does not already believe the premises and does not trust A concerning them. In this case, A can present a further argument for the premises or persuade B via some non-argumentative method, e.g. via demonstration.

These facts about the structure of argumentation can now be connected to Agrippa’s trilemma if reinterpreted, not as an argument for skepticism, but as one about argumentation *with a skeptic*. Suppose a speaker A intends to persuade a skeptical character S via argumentation who fulfills the following two conditions:

1. S does not trust A concerning any premise, i.e. S does not believe any premise merely because of A uttering it.
2. S is generally agnostic in that she does not believe any proposition (in a certain discursive domain) unless A presents an argument for it.

Both attitudes are not obviously epistemic vices, rather they have, at least at first sight, some rationale. Note that S is not generally stubborn in that she does not believe any proposition regardless of whether someone presents any argument for it. Rather, she does not believe any proposition unless one presents a persuasive argument. S’s attitude could be described as the general willingness to believe a proposition but only if one presents a persuasive argument for it. In this respect, S is only very cautious in forming her beliefs. Suppose now that A intends to persuade S via argumentation that *p*. S will only believe that *p* if A can persuasively argue for *p*. A can present an argument R for *p* consisting of premise *p*_1_.^25^ S will believe *p* based on *p*_1_ only if S also believes that *p*_1_. Due to S’s skeptical character, S will believe *p*_1_ only if A presents an argument for *p*_1_ consisting of premise *p*_2_. And so on.

We can see how argumentation with the skeptic leads to Agrippa’s trilemma. In what follows, I accept the premise that there are exactly three ways of arguing: Stopping the argumentation at some point, circular argumentation, and infinite argumentation. Let me investigate these three options with regard to argumentation with the skeptic. Take stopping at some point first. If A stops at some point arguing for *p* by uttering premise *p*_n_ but without presenting an argument for *p*_n_, then S will not believe *p*_n_ because of A’s utterance. Accordingly, S will not believe the conclusion *p*_n−1_ for which *p*_n_ is the premise and will eventually fail to believe that *p*. Hence, stopping at some point without providing further argumentation will not persuade the skeptic that *p*.

Let us next consider circular argumentation. Suppose that A uses circular argumentation, e.g. A argues for *p* via *p*_1_, for *p*_1_ via *p*_2_, and for *p*_2_ via *p*. If B does not believe *p* when A presents an argument based on *p*_1_ for it, then B still will not believe *p* when A uses it as a premise for arguing for *p*_2_, at least not if B is minimally coherent. Hence, A will not persuade B via circular argumentation, and accordingly A cannot persuade our skeptical character S via circular argumentation.

Only infinite argumentation remains. Usually, infinitism about argumentation is rejected by claiming that human beings have finite capacities and cannot provide infinite chains of argumentation.^26^ Therefore, it is concluded that we cannot persuade via infinite argumentation. This argument is sound—we cannot persuade anybody, including the skeptic, via infinite argumentation. However, the correct diagnosis of why we cannot persuade a skeptic via infinite argumentation is slightly different. Even if A were capable of providing an infinite chain of arguments, A would not persuade S via argumentation because A would never reach any premise that S believes and that can initiate the chain of persuasion. Hence, regardless of whether A can argue ad infinitum or not, she cannot persuade S via argumentation that *p*.^27^

We can summarize the results as follows. There are exactly three options of arguing for *p*. Stopping at some point, presenting a circular argument, and arguing ad infinitum. First, we cannot persuade a generally skeptical person S by stopping at a certain premise because S does not believe that premise. Second, we cannot persuade S via circular argumentation because circular argumentation is never persuasive (given that the hearer is minimally coherent). Third, we cannot persuade S via infinite argumentation because there is no common starting point. (The impossibility of infinite argumentation is not the problem.) Since there is no further option, we cannot persuade a generally skeptical person. This is the reinterpretation of Agrippa’s Trilemma as an argument about the limits of persuasive argumentation with a skeptic.

Let me now come to deep disagreement. I am interested in cases of deep disagreement that result from different beliefs that a speaker and a hearer have concerning phenomena such as the truth of premises, rationality of arguments, and reliability of sources. In this section, I will focus on beliefs about the truth of premises.^28^ Agreement about *p* requires that two parties have the same doxastic attitudes towards *p*. Hence, agreement is only reached via persuasive argumentation if the hearer also believes the proposition for which she argues. If a speaker persuades a hearer about a proposition that the speaker does not believe, then no agreement is reached. Accordingly, I focus hereinafter on speakers who are *honest* in that they only present arguments whose premises and conclusions they believe. Speakers are not necessarily honest. A speaker might have the intention to mislead a hearer or to make her believe a proposition on the basis of fallacious argumentation. However, I assume that most speakers are in most contexts honest and that dishonest speakers who present arguments based on premises that they do not believe or who argue for conclusions that they do not believe are an exception. Moreover, I will focus on unprejudiced hearers who fulfill condition PCB, i.e. who believe the conclusion of an argument because of the argument only if they believe (sufficiently many) premises. Again, hearers do not necessarily fulfill this condition.

Suppose A presents an argument R for *p* to B based on premise *p*_1_. Given that B is unprejudiced, she will not believe that *p* based on R unless B believes that *p*_1_. Here, two positive options are available. B already believes that *p*_1_ prior to being confronted with it, or B trusts A and believes that *p*_1_ because of A’s utterance of *p*_1_. If neither of these cases obtains, then A might eventually persuade B via argumentation that *p* if A can present a persuasive argument R’ for *p*_1_, based on premise *p*_2_ and so on. However, if A fails to reach a point where B already believes the premises that A uses or where B trusts A concerning the premises, then A will fail to persuade B, given B’s unprejudicedness. Hence, we can say:

Given that B is unprejudiced, A can persuade B via argumentation that *p* only if A presents a chain of argumentation such that, at some point B already believes the premises or B trusts A concerning some premises.

Let us now also take the attitudes of speakers into account. Suppose that A is honest and only presents arguments whose premises A believes:

Given that A is honest and that B is unprejudiced, A can persuade B via argumentation that *p* only if A and B agree about some premises prior to engaging in argumentation or if B trusts A concerning some premises that A presents.

If A and B do not agree about some premises prior to engaging in argumentation or B does not trust A concerning some premises that A presents, then A cannot persuade B via argumentation. In this case, they deeply disagree and they cannot overcome this deep disagreement via argumentation.^29^

These issues on deep disagreement are linked to Agrippa’s trilemma in the following way: Suppose that A is honest and that B is unprejudiced. Suppose further that A and B do not share any premises and that B does not trust A concerning any premises. A has exactly three options for arguing for *p*, stopping at some point, circular argumentation and infinite argumentation. A will not persuade B by stopping her argumentation at some point. Moreover, A cannot persuade B via circular argumentation. Finally, A is not capable of arguing ad infinitum, but even if A were capable, A would not persuade B via infinite argumentation. Hence, A cannot persuade B via argumentation. In this way, we can reconstruct Agrippa’s trilemma as an argument about the limits of persuasive argumentation given that an honest speaker and an unprejudiced hearer do not share any beliefs about premises prior to engaging in argumentation and given that the hearer does not trust the speaker about any premise used.

## Meta-regresses

We next come to a skeptical regress argument involving higher-order propositions. As a starting point, an iteration principle, which has some prima facie plausibility, such as the JJ-Principle or the KK-principle, is accepted as a premise of the skeptical argument:

**JJ-principle**

S is justified in believing that *p* only if S is justified in believing that she is justified in believing that *p*.

**KK-principle**

S knows that *p* only if S knows that she knows that *p*.^30^

Since JJ and KK are general principles, they also hold for beliefs in higher-level propositions. They imply that one is justified in believing that *p* only if one is justified in believing infinitely many meta-propositions or one knows that *p* only if one knows infinitely many meta-propositions.^31^ However, we can neither justifiedly believe nor know infinitely many meta-propositions. Therefore, we cannot know that *p*. These are skeptical meta-regress arguments. A meta-regress argument, which is based on the JJ-principle, can be spelled out in detail as follows:

**The meta-regress argument**

1. JJ-principle
2. Therefore, S is justified in believing that *p* only if S is justified in believing infinitely many different meta-propositions.
3. S cannot be justified in believing infinitely many different meta-propositions.
4. Therefore, S cannot be justified in believing that *p*.
5. S knows that *p* only if S is justified in believing that *p*.
6. Therefore, S cannot know that *p*.

An analogous skeptical argument can be formulated on the basis of the KK-principle. Concerning such meta-regress arguments, two questions arise: (1) Is the underlying iteration principle true? (2) Why shouldn’t we be capable of justifiedly believing or knowing infinitely many meta-propositions (at least implicitly)? In contemporary epistemology, these meta-regress arguments are usually regarded as unsound and rejected by rejecting the underlying iteration principles. Hence, the first question is usually answered in the negative. The second question might be answered by tacitly assuming that being justified or knowing implies having performed an *act* of justifying or reasoning. Accordingly, we are only justified in believing that *p* if we have justified *p* and if we have justified that we have justified *p* and so on. Since we cannot perform infinitely many acts of justifying or reasoning, we cannot justifiedly believe infinitely many meta-propositions. Without going into details, one can note that this assumption is controversial. If justification or knowledge is simply a positive epistemic status of a belief, then why should this status require having performed a certain act of justification or reasoning?

In which situations should we be in need to reason that a belief, proposition, or claim is justified in order to reach a certain epistemic goal? Again, we can find an answer when considering argumentation between two parties. Suppose that A presents argument R for *p* to B. One potential reason for why B might fail to believe that *p* because of R is that she does not regard R as a good or rational argument for *p*.^32^ Suppose now that B challenges A’s argumentation by rejecting that R is a rational argument for *p* or by suspending judgment about whether it is one. In response, A can present a meta-argument for the claim that R is a rational argument for *p* and so on. Suppose A presents an inductive argument that *a*_n+1_ is F based on the premises that *a*_1_…*a*_*n*_ are F. B can, for example, challenge this argument by stressing that cases *a*_1_…*a*_*n*_ being F are not sufficiently many cases for supporting that *a*_*n*+1_ is *F*, or more fundamentally by rejecting inductive arguments in general. Analogously, if S presents an abductive argument for *p*, then B can challenge that the argument is in fact an inference to the *best* explanation or S can reject abductive arguments in general. In both cases, A is forced to present a meta-argument for the rationality of the first-order argument in order to persuade B. We can see how, under certain circumstances, speakers in argumentation can be forced to enter meta-regresses of justification, namely when the speaker doubts or rejects the rationality of an argument presented.

Let us consider again an argumentation with a skeptic. Let us now take a different skeptical character into account, someone who is skeptical about the rationality of arguments and not about the truth of its premises. Such a skeptic is a character with the following two features:

1. S does not believe the conclusion of an argument because of the argument unless S believes that the argument is rational.
2. S suspends judgment about the rationality of an argument unless one presents a persuasive argument that the argument is rational.

The first feature is a reflective one, which ensures that a skeptic about rationality is persuaded by an argument only if she also believes that the argument is rational. The second guarantees agnosticism concerning the rationality of arguments. If the skeptic suspends judgment about its rationality or rejects it as a rational argument, she does not believe the conclusion because of the argument. A skeptic about rationality of arguments is not obviously irrational in that she rejects any argument as irrational. The first feature is reasonable and the second feature characterizes someone who is generally cautious about rationality of arguments and does not believe of any argument that it is rational unless one provides reasons for it.

We can now easily see how a speaker A is forced to enter a potentially infinite meta-regress of argumentation when aiming at persuading a skeptic S about rationality. Suppose A presents argument R for *p*. Since S does not believe that R is a rational argument for *p* until A presents a persuasive argument, A has to present a meta-argument R’ for the claim that R is rational and so on. Since there is no argument or meta-argument of which S believes that it is rational without having been provided persuasive argumentation, S will not believe of any argument that it is rational and, consequently, will not believe via argumentation that *p*. Hence, nobody can convince a skeptic via argumentation about any proposition who is generally agnostic about the rationality of arguments.

Again, the reason for the impossibility of persuasion is not that speaker A would have to undergo an infinite meta-regress of argumentation for persuasion which she is not capable of doing. Rather, agreement about the rationality of an argument as a common starting point is missing. Under these conditions, A would also not persuade S via argumentation even if she were capable of undergoing such an infinite meta-regress. This is the reinterpretation of skeptical meta-regress arguments as arguments about the limits of persuading a skeptic about rationality of arguments.

A skeptical character about the rationality of arguments is in disagreement with anybody who holds positive beliefs about the rationality of arguments. Let us now come to the more general case of deep disagreement between two parties about the rationality of arguments. In this paper, I am interested in speakers and hearers who follow at least their own standards of rational argumentation. For other subjects, there is hardly any systematic view available. Let us consider hearers who believe the conclusion of an argument because of the argument only if they believe that the argument presented is rational. Call such hearers *subjectively rational*. They are *subjectively* rational since they follow their own *beliefs* about rational arguments, which do not necessarily match with the facts.^33^

Suppose now that A presents argument R for *p* to B who is subjectively rational. If B does not believe that R is rational, she will not believe that *p* because of R. A has still the option to persuade B via a meta-argument MR that R is rational and thereby eventually persuade B that *p*, and so on. However, if there is no meta-argument which B believes to be rational, then A will eventually fail to persuade B that *p* via argumentation. Hence, it holds:

If B is subjectively rational and fails to believe that *p* because of R because B does not believe that R is a rational argument and there is no meta-argument MR* in any chain of meta-arguments for R such that B believes that MR* is a rational argument prior to being confronted with it by A, then A cannot persuade B that *p* via argumentation.^34^

Let me next come to deep disagreement. In order to investigate deep disagreement, we must also consider attitudes of speakers. Reflective speakers hold views about rational arguments. I call speakers who argue in line with their views in that they only present arguments that they believe to be rational *subjectively rational*. We can now state the following about single arguments:

If A and B are subjectively rational and disagree about the rationality of R, then A will not persuade B that *p* via R.

I assume that speakers and hearers are usually, though not always, subjectively rational.^35^ According to this assumption, it holds that usually, if A and B disagree about the rationality of R, then A will not persuade B that *p* via R.

So far, we have focused on disagreement about the rationality of single arguments. If a speaker and a hearer disagree about the rationality of an argument, then they can potentially settle the dispute by presenting a persuasive argument about the rationality of the first argument and so on. Accordingly, a regress of meta-arguments that is potentially infinite is possible. By considering not only single arguments but also meta-arguments and potential meta-regresses we can say:

If A and B are both subjectively rational and do not agree about the rationality of a single meta-argument MR* in any potential chain of meta-arguments for R prior to engaging in argumentation, then A cannot persuade B that *p* via argumentation.

I assume that speakers and hearers are usually subjectively rational. Consequently, if A and B generally disagree about the rationality of arguments, then usually A cannot persuade B that *p* via argumentation. This is an instance of deep disagreement, which is irresolvable via argumentation.

To sum up this section: We cannot persuade a skeptic via argumentation who is generally agnostic about the rationality of arguments. Moreover, usually, A can only persuade B via argumentation if A and B agree about the rationality of some arguments prior to engaging in argumentation. If A and B lack this kind of agreement, then they deeply disagree.

## Mooreanism

Let us now come to the argument for external world skepticism, which traces back to Descartes’ (1931) work and which is nowadays the most debated skeptical argument. Presumably, Mooreanism is currently the most controversial and most debated solution to the skeptical problem.^36^ In the contemporary literature, Mooreanism is usually interpreted as a theory about the architecture of knowledge. This version of Mooreanism, which is adopted here, is characterized by the following two claims:

**Mooreanism**

1. External world knowledge is epistemically prior to knowledge that the skeptical hypotheses are false.
2. We can know partly through competent inference from external world knowledge that the skeptical hypotheses are false.^37^

In order to make her point, the skeptic must explain why Moorean reasoning cannot yield knowledge. There are various explanations of the defectiveness of Moorean reasoning on the market, defended by skeptics but also by many anti-skeptics. The most influential explanations for the defectiveness of Moorean reasoning are:

**Anti-Moorean arguments**

Moorean reasoning is defective:

1. Because it cannot offer a persuasive argument to the skeptic;
2. Because it can only deliver the result that the skeptical hypothesis is false;
3. Because we must have additional and independent knowledge that the skeptical hypothesis is false.^38^

In this paper, I will focus on the first anti-skeptical argument. This strategy is discussed by Pryor (2004), who argues that Moorean arguments are “persuasively crippled,” but in terms of their justificatory structure are nothing to be ashamed of.^39^

Moorean reasoning can be understood as an instance of the more general phenomenon of bootstrapping. Bootstrapping, as introduced and discussed by Vogel (2000, 2008) is a process of reasoning for the reliability of a source based on information delivered by this very source.^40^ Vogel presents the example of Roxanne who comes to believe that her gas gauge is reliable by repeatedly looking at it. Here is a pattern for bootstrapping reasoning:

**Bootstrapping**

- P_1_: *p*_1_.
- P_2_:* O* indicates that *p*_1_.
- P_3_:* p*_1_ ∧ (*O* indicates that *p*_1_).
- Repeat for *p*_2_…*p*_n_.
- C:* O* is a reliable source.^41^

Bootstrapping is, like Moorean reasoning, intuitively an epistemically defective procedure, and one way of explaining its defectiveness is by means of its persuasive defectiveness. Intuitively, bootstrapping is a flawed method of persuading someone that a source is reliable. For example, if B doubts that a gas gauge is reliably working, then A will hardly convince B about its reliability by pointing towards its indications and by performing bootstrapping reasoning.

There is wide agreement among contemporary epistemologists that justification and knowledge do not suffer from any of the two regress problems discussed above and that, therefore, these arguments are unsound. As for Mooreanism and bootstrapping, the situation is different. In fact, there is an ongoing discussion about whether S can justifiedly believe or know via Moorean reasoning or bootstrapping.^42^ Accordingly, this skeptical problem is currently regarded as more serious than Agrippa’s trilemma and the meta-regress argument.

Let us see in more detail why bootstrapping and Moorean reasoning are not instances of persuasive argumentation. Bootstrapping argumentation roughly involves a speaker A demonstrating that source *O* indicates that *p* (and perhaps thereby uttering that *p*), uttering that *O* indicates *p*, and then uttering the further inferences required for bootstrapping.^43^ The persuasive defectiveness of bootstrapping can be explained by relying on two assumptions. First, I will accept principle PCB for B, that B believes the conclusion of arguments because of the argument only if B also believes its premises (or at least sufficiently many of them). Second, I will consider hearers for whom the following principle holds:

**DR**

If B rejects that source *O* is reliable or suspends judgment about *O*’s reliability and believes that information *i* is delivered by *O*, then B suspends judgment about the truth of *i*.^44^

I assume here that S does not have any further evidence about the truth of *i*. It is important to note that DR expresses a rational behavior. Given that S rejects that *O* is reliable or suspends judgment about its reliability, it is rational for S to suspend judgment about whether information delivered by *O* is true. For example, it is irrational for a subject to believe that a certain thermometer is unreliable but still believe what the thermometer indicates on the basis of its indication. Rather it is rational to suspend judgment about the truth of the indication. The rationality involved here is a kind of internal coherence of the subject’s body of beliefs, which must not be confused with doxastic or propositional justification. *Given* that S doubts that *O* is reliable, it is rational for S to suspend judgment about the truth of information delivered by *O*. This doubt however, might be justified or not.^45^

Putting the pieces together, we can now formulate an argument for why bootstrapping is not a persuasive way of arguing.

**Argument for the unpersuasiveness of bootstrapping**

1. Suppose that B rejects that source *O* is reliable or suspends judgment about O’s reliability.
2. B believes that *c* because of A’s argumentation consisting of premises *p*_1_…*p*_n_ and conclusion *c* only if B believes sufficiently many premises *p*_1_…*p*_n_. (PCB)
3. If B rejects that *O* is reliable (or suspends judgment about *O*’s reliability) and believes that information *i* is delivered by *O*, then B suspends judgment about the truth of *i*. (DR)
4. In case of bootstrapping understood as argumentation, information delivered by *O* constitutes the premises of an argument that* O* is reliable. (Definition of bootstrapping argumentation)
5. B *usually* recognizes A’s bootstrapping argumentation about *O* as bootstrapping argumentation.^46^
6. Therefore, A usually does not persuade B that *O* is reliable via bootstrapping.

Take Vogel’s example of the gas gauge *G*. Suppose that B suspends judgment about the reliability of *G*. If A wants to persuade B via bootstrapping argumentation that *G* is reliable, then this project will fail. B does not believe the indications of *G*, since she suspends judgment about the reliability of *G*, and, consequently, S does not believe the conclusion of the bootstrapping argument.

Let us now investigate the particular case of Moorean reasoning. With Moorean reasoning, the process of argumentation is not one between two actual persons. Rather, it must be understood as an argumentation between an inner believer and an inner skeptic.^47^ We can explain why Moorean reasoning is not an instance of persuasive argumentation of the claim that one’s sense apparatus is reliable as follows:

**Moorean argumentation**

Suppose S’s inner skeptic B doubts that S’s sense apparatus is reliable and A, S’s inner believer, aims to persuade B about its reliability via Moorean argumentation.

1. A demonstrates that S’s experience delivers that *p* by pointing towards S’s experience
2. B thereby believes that S’s sense apparatus delivers that *p*
3. A utters that *p*
4. B suspends judgment about whether *p* is true based on S’s experience as of *p* (DR)
5. Therefore, B does not believe that S’s experience accurately indicates that *p* (PCB)
6. …
7. Therefore, B does not believe via Moorean argumentation that S’s sense apparatus is reliable

This is the reinterpretation of the argument for external world skepticism including the claim that Moorean reasoning is epistemically flawed for it is not persuasive as an argument about the limits of argumentation.

Let us now consider the case of arguing with a skeptic S, not necessarily an inner one, who is generally agnostic about the reliability of sources and suspends judgment about the reliability of any source unless one provides a persuasive demonstration for its reliability.^48^ If B doubts that source *O* is reliable then A can use another source *O’* for persuading B that *O* is reliable. For example, A can use a dipstick for demonstrating that a gas gauge is reliable. However, A will succeed in persuading B that *O* is reliable via *O’* only if B believes that *O’* is reliable, and so on. This way, problems concerning Moorean reasoning can collapse into an infinite regress problem.^49^ We can now see why a speaker cannot persuade a skeptic who is generally agnostic about the reliability of any source. Such a skeptic, ex hypothesi, doubts the reliability of any source that a speaker can use, and, consequently, the speaker cannot persuade the skeptic about the reliability of any source and about any proposition by using a particular source.^50^

This analysis of the limits of argumentation with skeptics about reliability is of rather theoretical interest. Let us now investigate the consequences for deep disagreement, which is a broader and more relevant social phenomenon. By generalizing the argument for the impossibility of persuading a skeptic who is skeptical about the reliability of sources and assuming that B follows DR we can say:

If B doubts the reliability of any source that A can use for delivering premises for an argument for *p*, then A cannot persuade B via a source that *p*.

Suppose now that A is honest about sources in that she uses sources for argumentation only if A believes that these sources are reliable and B follows DR. In this case, it holds:

A can only persuade B via a source that *p* if A and B agree about the reliability of a source prior to engaging in argumentation.

If A and B lack any agreement about the reliability of sources that A can use in order to argue for *p*, then A fails to persuade B that *p* for a systematic reason. In this case, speaker and hearer deeply disagree and this disagreement cannot be resolved by consulting any source.^51^

## Conclusion

Some skeptical arguments have a certain intuitive appeal, but they are not sound arguments about the extent of knowledge. However, they can be reinterpreted as arguments about the preconditions and limits of persuasive argumentation, explaining why we cannot persuade skeptical characters. Moreover, these reinterpretations reveal certain instances of deep disagreement. (1) A reinterpretation of Agrippa’s trilemma shows that we cannot persuade a skeptic who is agnostic about any proposition by stopping at a certain point, circular argumentation, or infinite argumentation. Two parties deeply and irresolvably disagree if they do not share any agreement about sufficiently many premises prior to engaging in argumentation. (2) Skeptical meta-regress arguments can be reinterpreted as showing that we cannot persuade a skeptic who is generally agnostic about the rationality of arguments. There is deep and irresolvable disagreement between two parties who do not share any agreement about the rationality of arguments prior to engaging in argumentation. (3) Bootstrapping and Moorean reasoning are not instances of persuasive argumentation. Two parties deeply and irresolvably disagree if they do not agree about the reliability of any source prior to engaging in argumentation.^52^

One might object that skepticism enters through the backdoor when reinterpreting skeptical arguments as arguments about the limits of argumentation with the skeptic since the fact that one cannot provide a persuasive argument to the skeptic for *p* shows that one does not know that *p*. However, this line of thought is mistaken. The insights about the corresponding versions of deep disagreement show that the capacity of persuading someone via argumentation is determined by whether speaker and hearer share sufficiently many beliefs about the truth of premises, rationality of arguments, or reliability of sources. It does not depend on the epistemic position of the speaker. A speaker might be in a perfect epistemic position concerning a proposition, i.e. she might know the proposition according to the highest standards for knowledge, yet she might fail to persuade a skeptic or someone who does not share the beliefs required for persuasive argumentation. Therefore, knowing that *p* or being justified to believe that *p* does not imply having the capacity to persuasively argue for *p*. The skeptical move of pointing towards the persuasive defectiveness of certain arguments and then concluding that one cannot know or be justified to believe is illegitimate.^53^

As for deep disagreement, two questions are of particular interest: What is deep disagreement? Is deep disagreement resolvable or not? Both questions have been settled. We see that there are at least three kinds of deep disagreement, one relying on systematic disagreement about premises, one about the rationality of arguments, and one about the reliability of sources.^54^ These versions of deep disagreement actually are irresolvable. Hence, there is not one single type of deep disagreement. Deep disagreement is a heterogeneous phenomenon.^55^ The question about the nature of deep disagreement is in this sense misleading. Moreover, at least for these types of deep disagreement discussed here pessimists about the resolvability are right.

The theses about the limits of persuasive argumentation with a skeptic are of rather theoretical interest, providing a new view on well-known skeptical arguments. However, the theses about variants of deep disagreement and the explanation for their irresolvability are of significant social relevance. Actual instances for any type of deep disagreement can presumably be found, for example, concerning the preconditions of intercultural argumentation. However, finding and discussing such applications is beyond the scope of this paper.

## Footnotes

1 See Hill (1996), Lehrer (1999), Pryor (2000, 2004), and Markie (2005). For a recent attempt to present an argument against skepticism that is persuasive according to the skeptic’s standards, see Rinard (2018).

2 For epistemological reflections on argumentation, see Feldman (1994, 1999) and Goldman (1994, 1999). For epistemological argumentation theories, see Biro and Siegel (2006), Siegel and Biro (1997, 2008), Godden (2010) and Walton (2013).

3 Argumentation theory is understood by its proponents to be “an interdisciplinary study in its own right with logical, dialectical, and rhetorical dimensions that are to be nourished by the combined efforts of philosophers, logicians, linguists, discourse analysts, communication scientists, rhetoricians, psychologists, lawyers, and all others who have something to contribute that is theoretically pertinent” (Van Eemeren et al., 2014, p. 29). For an overview of the epistemological branch of argument theory, see Lumer (2005). For an overview of argumentation theories that approach argumentation from different interdisciplinary angles, see van Eemeren et al. (2014).

4 See van Eemeren and Grootendorst (2004, 60f).

5 I will discuss these different explanations of why Moorean reasoning is or seems flawed below. Moorean solutions to the skeptical problem accept the closure principle for knowledge but turn it against the skeptic when arguing that knowledge that the skeptical hypotheses are false can be inferred from external world knowledge. However, it is disputed whether all kinds of arguments for external world skepticism, including versions based on intuitions of indiscriminability and underdetermination, and solutions to these skeptical problems, rely on a closure principle. For a discussion see Cohen (1998) and Pritchard (2005).

6 For this presentation, see Wieland (2014). For an overview of the literature on Carroll’s puzzle, see Imholtz and Moktefi (2016).

7 In this respect, Carroll’s reconstruction of (A2) is superficial. For a discussion of the structure of regress arguments, see Wieland (2014).

8 Moreover, some authors establish a connection between arguments (A1)–(A3) and the problem of the criterion, as influentially discussed by Chisholm (1973). Cling (2009, 2014) argues that regress problems and the problem of the criterion are both instances of the same problem type, namely they are “paradoxes of reasons”. Aikin (2021a) argues for a close connection between the problem of the criterion, infinite regresses, and deep disagreement. Sosa (2007) establishes a connection between the problem of the criterion and (A3). For an overview on the problem of the criterion see McCain (2021). The situation concerning the problem of the criterion is not entirely clear. In fact, it is disputable whether a single version of the problem can be extracted or whether different problems must be distinguished. In this paper, I will leave open whether the insights gained about (A1)–(A3) are also applicable to the problem of the criterion.

9 Moreover, having beliefs of different strength or having different credences can be regarded as an instance of disagreement. In this paper, I will focus on the orthodox form of disagreement, since it matches with our verbal behavior of affirming or rejecting propositions, which is crucial for argumentation. However, I think that similar results can be obtained for a more fine-grained conception of doxastic attitudes.

10 This definition is meant to exclude deviant cases of causing a belief that *p*, e.g. via the mere melodiousness of an argument presented. Such deviant cases could be explicitly excluded by requiring in addition that *c* involves a specific content, e.g. *c* is an assertion that *p* or an argument for *p*, and that B believes that *p* because of the correctly understood content of *c*.

11 Page numbers refer to reprinted version in *InformaI Logic* 25(1), (2005), 3–11.

12 Kusch (2021) contains an extensive discussion of Wittgensteinian approaches to deep disagreement.

13 Ranalli (2021) distinguishes between a Wittgensteinian theory or hinge theory of disagreement and a fundamental epistemic principle theory.

14 Wittgenstein (1969) and some Wittgensteinians argue that there are some fundamental propositions, so called hinge propositions, that we not only cannot rationally justify but which we cannot rationally assess at all. If we follow Wittgenstein, rational doubt about hinge-propositions is not only unmandated, it is simply impossible (See Pritchard 2016.) In this paper, I will argue for the pessimistic view that there are various forms of disagreement with a skeptic that cannot be resolved via argumentation. When taking the Wittgensteinian approach into account, we can actually distinguish two types of pessimists concerning the rational resolution of deep disagreement with a skeptic, one that assumes that deep disagreement cannot be rationally resolved, and an even stronger one, based on hinge epistemological considerations, that rational responses to deep disagreement are generally impossible. For a critical assessment of hinge epistemology in the context of deep disagreement, see Siegel (2021).

15 For varying interpretations of the problem, see Pryor (2013). For a formulation of the trilemma that is based on defeasibility, see Klein (2008, p. 485).

16 Foundationalism is by far the most popular solution to Agrippa’s trilemma. Externalist theories of knowledge and justification are paradigmatically foundationalist and most internalist theories are foundationalist as well. For internalist foundationalist accounts, see Pryor (2000, 2004) and Huemer (2000, 2001). Coherentism does not really defend the view that we can justify beliefs via circular reasoning. Rather it claims that a belief is justified by being a member of a coherent set of beliefs where the beliefs mutually support and justify each other. See BonJour (1985) and Lehrer (1999, 2000). Infinitism, as proposed by Klein (1999, 2003, 2007), is a rather novel take on Agrippa’s trilemma. In this paper, I will not discuss these views about knowledge and/or justification. Representatives of argumentation theory also discuss Agrippa’s trilemma, albeit often with a different aim. By referring to critical rationalism as defended by Popper (1959), van Eemeren and Grootendorst (1984) accept the conclusion from Agrippa’s trilemma that there is no positive epistemic justification for arguing for their own concept of dialectical reasonableness. For a criticism of their straightforward adoption of this epistemically highly controversial skeptical view, see Siegel and Biro (2008).

17 One alternative interpretation has it that Agrippa’s trilemma concerns the status of someone’s assertions instead of someone’s beliefs. According to this take, S’s assertion that *p* is justified (or legitimate) only if S can provide reasons for it. However, this interpretation seems implausible. Intuitively, S’s assertion that she has a headache is justified or legitimate even if she cannot provide any further reasons in support of this claim. How could she? Hence, Agrippa’s trilemma reformulated about the justification or legitimation of assertions is not promising.

18 Note that persuasion only involves belief formation in a hearer. Hence, persuasion must be distinguished from issues about transmission of justification or knowledge on the basis of trust. If an utterance not only causes a belief but also creates knowledge, then we have an instance of testimonial knowledge. In this paper, I am more interested in belief-forming processes than in testimonial knowledge.

19 These types of belief formation might not be properly labelled ‘persuasion.’ However, I am concerned about belief forming processes via argumentation which are plausibly regarded as instances of persuasion. Hence, I use ‘persuasion’ in a wider sense.

20 I talk here about utterances rather than about assertions because a speaker can persuade a hearer also about a proposition that the speaker does not believe and, therefore, does not assert.

21 Hearers can believe what speakers utter for various reason, e.g. for their charisma. This can also be the case if the speakers present an argument. For example, a hearer might believe a conclusion merely because of the sound of the argument. I want to understand the provided definition of persuasion via argumentation in a sense that in such deviant cases the hearer does not believe the speaker *because of* the presented argumentation.

22 For the sake of simplicity, I will assume hereinafter that any argument contains exactly one conclusion.

23 In this paper, I am concerned with speakers and hearers who fulfill minimal criteria of rationality and utter arguments and believe conclusions of arguments at least according to their own standards of rationality. I regard hearers as minimally rational only if they do not violate PCB. I argue that subjects who fulfill these minimal criteria of rationality can fail to reach agreement under certain circumstances. However, for being an actually rational hearer, further requirements must be fulfilled, for example, that B also believes the premises *rationally* by her own lights. However, the overall results about disagreement remain the same if such further conditions are added.

24 PCB does not claim that the act of argumentation is only a vehicle for presenting an argument and that the hearer believes the conclusion of the argument merely because of the argument presented. It also plays a crucial role, whether the hearer trusts the speaker concerning the premises and believes the premises because of A’s uttering them.

25 I assume here, for the sake of simplicity, that A’s arguments consist of only one premise. However, the outcome is the same if we assume that A presents arguments with more premises. Moreover, one can think of the single premise simply as the conjunction of several premises.

26 This view about actual argumentation is also accepted by infinitists, who defend infinitism for justification.

27 For this point, see Ginet’s (2005) criticism of infinitism.

28 Deep disagreement results from a lack of common ground between speaker and hearer that renders persuasive argumentation impossible. The failure to persuade a skeptic results from a lack of common ground between the skeptic and any speaker since they do not share any premise and the skeptic does not trust the speaker.

29 One might object that not all arguments require affirmation of the premises, for example reductio arguments are solely based on supposition but not on affirmation of the premises. However, reductio arguments are usually embedded in a larger context that involves affirmation of the premises and of the conclusion based on it. For example, a reductio argument takes the complete form: (1) If *p* is true, then *q* is true. (2) *q* is false. (3) Therefore, *p* is false. In this case, the premises and conclusion are both affirmed. I assume that other forms of arguments that also seem not to involve affirmation of premises can be reformulated analogously. Zarefsky (2011), for example, suggests various strategies for resolving deep disagreement by embedding it in such larger contexts. However, he considers rather practical real-life cases. The strategies he suggests are not applicable to the more radical skeptical cases discussed here.

30 A version of the JJ-principle for idealized inferential justification is defended by Fumerton (1995, 2015). For discussions on iteration principles, see Greco (2014, 2015a, b).

31 Here it is tacitly assumed that self-referring higher-level beliefs are not justified. For the opposite view, see Lehrer (1999).

32 I use here the notion of rational arguments as a technical term referring to arguments where the truth of the premises supports the truth of the conclusion in an appropriate way. For the purpose here, I can leave open which arguments actually are rational.

33 Notably, the skeptic about the rationality of arguments is also subjectively rational. In this respect, her behavior in argumentations has some prima facie rationality.

34 I ignore here the option that B trusts A concerning the rationality of arguments in that B believes that R is a rational argument merely because A presents R. I assume that these cases of trust are possible but less usual than trust about premises.

35 For example, speakers might present arguments that they regard as irrational with the aim to deceive hearers, and hearers might fall prey to wishful thinking and believe conclusions of arguments because of the argument although they do not believe that the argument is rational.

36 Moore (1959) accepts the premise that S has knowledge of the external world only if S knows that the skeptical hypothesis is false, but highlights that it “cuts both ways.” He uses this premise and the claim that we have external world knowledge as premises for concluding that we know that the skeptical hypotheses are false. Strictly speaking, this argument tells us only *that* we know that the skeptical hypotheses are false but not *how* we know it. See Neta (2008). For a defense of Mooreanism, see Pryor (2000). Dretske (2005), in contrast, claims that Moore’s proof sounds like chutzpah and not philosophy to him.

37 According to this version of Mooreanism, external world knowledge is epistemically prior to knowledge that the skeptical hypothesis is false. One might agree with this version of Mooreanism that anti-skeptical knowledge is not a pre-condition for external world knowledge but also assume that it is a necessary condition because whenever one has external world knowledge, then one knows via competent inference from this knowledge that the skeptical hypothesis is false (or is at least in a position to know that.) I cannot provide here an exegetical discussion about what the correct version of Mooreanism is. Notably, this stronger version rules out the possibility of having external world knowledge without knowing that the skeptical hypothesis is false. However, an infant plausibly has the capacity to know that there is a red ball in front of her, but does not have the cognitive and conceptual capacity to know that she is not a systematically deceived brain in a vat. For the purposes of this paper, I need not commit myself to this stronger version of Mooreanism.

38 Sensitivity theorists like Nozick (1981) choose the second strategy. A similar strategy is suggested by Titelbaum (2010) and Douven and Kelp (2013), who argue that bootstrapping cannot yield knowledge because it could never yield a negative result. Weisberg (2012) calls this the *no risk, no gain diagnosis*. This strategy is also adopted in Melchior (2019) where I argue that due to its insensitivity Moorean reasoning is not a method for checking, while leaving open whether it is a method of knowing. The third road is taken by Wright (2002, 2004), who claims that Moore’s argument fails to be cogent since warrant does not transmit from the premises to the conclusion. For a criticism of Wright, see Pryor (2004).

39 See also Hill (1996) and Lehrer (1999, 2000) for such a criticism. I argue in Melchior (2012) that Lehrer’s coherentism and Mooreanism both face problems of unpersuasiveness.

40 Issues about bootstrapping are also discussed under the label “epistemic circularity.” See Alston (1986) and Lynch (2010).

41 Bootstrapping reasoning can also lead to other conclusions, e.g. that all deliverances of O are true or that O is not unreliable (for reason R). In this paper, I will focus on the instance of bootstrapping to the conclusion that a source is reliable. However, similar results can be obtained for other instances of bootstrapping. See Melchior (2016).

42 For an overview, see Weisberg (2012).

43 So far, we have focused on argumentation that merely involves premises in support of a conclusion. Since this process also involves *demonstrations* that *p* via *O*, the framework for argumentation used so far must be slightly extended.

44 They might also believe that the information is false if they not only fail to believe that the source is reliable but also believe that it systematically provides false information.

45 For this distinction between justified beliefs and what is rational to believe given certain beliefs, see Pryor (2004).

46 This is at least true for paradigmatic cases of bootstrapping such as Vogel’s gas gauge case or Moorean reasoning, which are discussed in the literature. They are *obviously* instances of bootstrapping.

47 Suppose Moorean argumentation is reconstructed as an argumentation between two real persons, A and B. B doubts that the external world is broadly the way she experiences it to be. In this case, B plausibly also doubts that A actually exists and that the argumentation is A’s argumentation. B would take the dialogue to be a kind of inner dialogue. However, this is not the intuitive problem of Moorean argumentation. Hence, it has to be reconstructed as an inner dialogue.

48 One might argue that a skeptic who is agnostic about the reliability of any source pursues a project that is, to a certain extent, self-undermining, since the skeptic should also be agnostic about her own reasoning for being agnostic. Hence, the position of being agnostic about every source but having come to believe this position via reasoning is self-refuting. In this case, trying to persuade such a skeptic means to persuade someone who is already irrational. Moreover, Rinard (2018) has argued that accepting external world skepticism commits one to the self-undermining position of rejecting on the basis of complex reasoning that complex reasoning is a source of knowledge or justification. However, these issues about the incoherence of the skeptical position are not a pressing problem for the line of augmentation provided here. The skeptic might not have become agnostic via reasoning concerning all sources. Rather, we can easily imagine a skeptic who is agnostic about all sources from the very beginning or who practices agnosticism as a kind of habit. Such a character is, of course, idealized and hypothetical, but this holds for any kind of global skeptic.

49 Nevertheless, it useful to keep problems of Moorean reasoning and infinite regresses apart. Accepting Moorean reasoning as a source for transferring justification or knowledge is one way of avoiding this particular version of an infinite regress argument, but it is not necessary. Conservatives about Moorean reasoning such as Wright (2002, 2004) reject Moorean reasoning, nevertheless they also reject skepticism by assuming that we have a kind of a priori justification or entitlement to believe that our sense apparatus is reliable.

50 I assume here that A herself is also a source and, therefore, S does not trust A concerning her assertions unless A persuasively demonstrates her reliability.

51 Lynch (2010) presents the case of two subjects who deeply disagree because A believes that the holy bible as a source overrides any other evidence while B rejects this view.

52 Some proponents in argumentation theory, for example Johnson (1996), argue that arguments not only have a logical tier, which concerns the supporting relation between premises and conclusion, but also a dialectical tier, which concerns objections and alternative positions. According to a radical interpretation of this dialectical tier, an argument is proper only if it addresses all possible objections and alternative positions. Such a strong requirement obviously leads to an infinite regress, independent of a skeptical hearer to whom the argument is presented. On this point, see Govier (1998).

53 Externalist but also many internalist views of justification have it that S can be justified in believing that *p* without being in a position to publicly present a reason for their belief. This is obviously the case for externalists, for example reliabilism, since a belief can be justified by being caused by a reliable process even if the believing subject does not have access to this fact. However, Pryor (2000, p. 520), an internalist, also proposes in a similar spirit that “not only can we have perceptual knowledge and justified perceptual belief, we might have it without being in a position to cite anything that could count as ampliative, non-question-begging evidence for those beliefs.” Dialecticalists such as Brandom (1994), Leite (2004, 2005), and Williams (2003) diverge from this mainstream view when holding that “the norms of well-run critical dialogue are the norms of justification, so the justificatory status of our beliefs depends on how we are able to defend them in the face of criticism” (Aikin, 2021b, p. 269). Leite (2004, p. 245), for example argues “that the justificatory status of our beliefs is determined by what we are able to do when we attempt to defend our beliefs.” Moreover, some dialecticalists also discuss characters that are similar to the skeptical characters discussed here, for example Leite’s (2005) ‘persistent interlocutor’. See also Rescorla (2009) who argues against dialecticalists for a structural distinction between defending one’s view against opponents and being justified to believe. These authors make valuable contributions on the relation between being justified and being able to provide a reason, but they address a different phenomenon than this paper. There is a subtle but crucial difference between defending one’s view against objections and persuading someone. A can defend her position against objections according to the highest standards of reasonability, yet nevertheless fail to persuade B, for example, if B is a skeptic about all premises or about the rationality of all arguments presented or about the reliability of all sources. I leave a more substantial reflection on the asymmetries of reasoning for persuasion and for defending one’s view for another occasion.

54 Lynch (2010, p. 267) claims that, where deep epistemic disagreement occurs, then the disagreement is ultimately disagreement over fundamental epistemic principles, which apparently concern reliability of sources and principles such as induction or abduction. We have seen that this is not correct, since deep disagreement can also result from fundamental disagreement about the reliability of sources, which does not concern any fundamental epistemic principle.

55 Moreover, the list of instances of deep disagreement is presumably not exhaustive. Other versions, e.g. based on conceptual incommensurability presumably exist.
